# Expression-based decision tree model reveals distinct microRNA expression pattern in pediatric neuronal and mixed neuronal-glial tumors

**DOI:** 10.1186/s12885-019-5739-5

**Published:** 2019-06-06

**Authors:** Magdalena Zakrzewska, Renata Gruszka, Konrad Stawiski, Wojciech Fendler, Joanna Kordacka, Wiesława Grajkowska, Paweł Daszkiewicz, Paweł P. Liberski, Krzysztof Zakrzewski

**Affiliations:** 1 Department of Molecular Pathology and Neuropathology, Medical University of Lodz, Pomorska 251, 92-216 Lodz, Poland; 2 Department of Biostatistics and Translational Medicine, Medical University of Lodz, Mazowiecka 15, 92-215 Lodz, Poland; 3 Department of Radiation Oncology, Dana-Farber Cancer Institute, Boston, MA USA; 4 Department of Pathology, The Children’s Memorial Health Institute, Av. Dzieci Polskich 20, 04-730 Warsaw, Poland; 5 Department of Experimental and Clinical Neuropathology, Mossakowski Medical Research Centre, Pawinskiego 5, 02-106 Warsaw, Poland; 6 Department of Clinical Department of Neurosurgery, The Children’s Memorial Health Institute, Av. Dzieci Polskich 20, 04-730 Warsaw, Poland; 7 Department of Neurosurgery, Polish Mother Memorial Hospital Research Institute in Lodz, Rzgowska 281/289, 93-338 Lodz, Poland

**Keywords:** Brain tumor, Differentiation model, Dysembryoplastic neuroepithelial tumor, Expression, Ganglioglioma, microRNA, Neuronal and mixed neuronal-glial tumor, Pediatric, Pilocytic astrocytoma

## Abstract

**Background:**

The understanding of the molecular biology of pediatric neuronal and mixed neuronal-glial brain tumors is still insufficient due to low frequency and heterogeneity of those lesions which comprise several subtypes presenting neuronal and/or neuronal-glial differentiation. Important is that the most frequent ganglioglioma (GG) and dysembryoplastic neuroepithelial tumor (DNET) showed limited number of detectable molecular alterations. In such cases analyses of additional genomic mechanisms seem to be the most promising. The aim of the study was to evaluate microRNA (miRNA) profiles in GGs, DNETs and pilocytic asytrocytomas (PA) and test the hypothesis of plausible miRNA connection with histopathological subtypes of particular pediatric glial and mixed glioneronal tumors.

**Methods:**

The study was designed as the two-stage analysis. Microarray testing was performed with the use of the miRCURY LNA microRNA Array technology in 51 cases. Validation set comprised 107 samples used during confirmation of the profiling results by qPCR bioinformatic analysis.

**Results:**

Microarray data was compared between the groups using an analysis of variance with the Benjamini-Hochberg procedure used to estimate false discovery rates. After filtration 782 miRNAs were eligible for further analysis. Based on the results of 10 × 10-fold cross-validation J48 algorithm was identified as the most resilient to overfitting. Pairwise comparison showed the DNETs to be the most divergent with the largest number of miRNAs differing from either of the two comparative groups. Validation of array analysis was performed for miRNAs used in the classification model: miR-155-5p, miR-4754, miR-4530, miR-628-3p, let-7b-3p, miR-4758-3p, miRPlus-A1086 and miR-891a-5p. Model developed on their expression measured by qPCR showed weighted AUC of 0.97 (95% CI for all classes ranging from 0.91 to 1.00). A computational analysis was used to identify mRNA targets for final set of selected miRNAs using miRWalk database. Among genomic targets of selected molecules *ZBTB20*, *LCOR*, *PFKFB2*, *SYNJ2BP* and *TPD52* genes were noted.

**Conclusions:**

Our data showed the existence of miRNAs which expression is specific for different histological types of tumors. miRNA expression analysis may be useful in in-depth molecular diagnostic process of the tumors and could elucidate their origins and molecular background.

**Electronic supplementary material:**

The online version of this article (10.1186/s12885-019-5739-5) contains supplementary material, which is available to authorized users.

## Background

Pediatric neuronal and mixed neuronal-glial tumors represent about 10% of all tumors of central nervous system and in the large number of cases are characterized by slow growth and favorable outcome [1]. The frequent manifestation of these relatively rare brain lesions is tumor-related epilepsy responsive to neurosurgical treatment. The taxonomy of the glioneuronal tumors evolved immensely during last two decades; the current WHO classification encompasses 13 entities with still the most frequent dysembryoplastic neuroepithelial tumor (DNET) and ganglioglioma (GG). The classification system changes are the effects of advantages both in histopathological and molecular analyses, however, pediatric low grade tumors lack the multiple genomic alterations observed in higher grade lesions [1–3]. In view of emergent data concerning molecular background of brain tumors further modifications cannot be excluded, especially that understanding of the origin and etiology of neuronal and mixed neuronal-glial tumors may evolve [3–5].

Coexistence of glial and glioneuronal compartment within the tumors as well as their possible histological transformation was an impulse to try to elucidate them in our analysis [6–10]. Recent evidences suggest that in brain tumors, similarly as in the other type of cancer, regulatory aspects of genomic machinery could be essential for tumor pathogenesis, progression and outcome. One of such promising factors is microRNA (miRNA) considered to be one of the crucial transcription modifiers associated with the development of cancer. miRNA analyzes could bring useful and valuable data, especially that only a limited number of structural alterations were observed in the neuronal and mixed neuronal-glial tumors [11–13].

Analysis of such molecular events could also be incorporated into the modern multilayered tumor diagnosis, especially that the miRNA expression seems to be more closely correlated with tumor origin and stage than mRNA status [14, 15]. Nonetheless, the significance of miRNAs expression in rare human disorders remains frequently unclear.

The aim of our study was to elucidate the potential relevance of miRNA expression in glial and glioneuronal pediatric brain tumors. According to that, we performed comprehensive two-stage analysis of miRNA expression in a representative group of those relatively rare pediatric brain tumors with enigmatic molecular background.

## Methods

### Patients and tumor samples

This prospective cohort analysis consisted of a total number of 148 children with low-grade tumors (Table 1, Additional file 1: Table S1).

**Table 1:** The summary of the clinicopathological features of samples analyzed in the study

Variable	*N* = 148
Age (years)	
Median (range 0.5–18)	9
Gender	
Male (%): Female (%)	52: 48
WHO classification	
Pilocytic astrocytoma	45
Dysembryoplastic neuroepithelial tumor	46
Ganglioglioma	57
Location	
Supratentorial	118
Infratentorial	30
Recurrence	4
Treatment	
Surgery	148

The group included patients with the diagnosis of low-grade tumor confirmed by histopathology report (DNET, GG, PA), thus surgically treated according to the standard of care guidelines. All patients were under 18 years of age at the time of surgery. Written informed consent was obtained from all examined individuals. Written informed parental consent was obtained for all patients under 16. For children below 16 years of age the consent was given by parents, for older informed consent was obtained from both parents and child, according to the Polish. The protocol of the study was approved by the Bioethical Committee at the Medical University of Lodz (permit No: RNN/63/14/KE).

### Isolation of miRNAs, RNA extraction and cDNA synthesis

Total RNA, including miRNA fraction was extracted from the tumor tissues stabilized in RNAlater and stored at − 80 °C until further processing. Samples were processed using a miRNeasy Mini Kit (Qiagen GmbH, Hilden, Germany) in accordance with the manufacturer’s protocol. Briefly, samples were incubated at room temperature for a period of 5 min with 700 μl of qiazol lysis reagent. After adding 140 μl of chloroform, the samples were shaken vigorously for 15 s, incubated at room temperature for 2 min, and centrifuged for 15 min at 4 °C. The upper aqueous phase, containing RNA was transferred to a new microcentrifuge tube and mixed with 1.5 volume of 100% ethanol, transferred to a spin column, centrifuged, washed, and eluted in 30 μl RNase-free water.

The quality and quantity of the samples were verified by standard electrophoresis and spectrophotometry methods, with an RNA integrity number (RIN) value ≥7 and ≥ 3 as a cut-off value for microarray and qPCR analysis respectively. For conversion of mature miRNA into cDNA the reverse-transcription reaction mix with Universal cDNA Synthesis Kit (Exiqon, Denmark) was prepared. Each cDNA was stored at − 20 °C until further analysis.

### MiRNA array analysis

The microarray experiment using the Exiqon platform was performed by Exiqon (Denmark). Briefly, 0.75 μg of total RNA from samples were labeled using the miRCURY LNA™ microRNA Hi-Power Labeling Kit, Hy3™/Hy5™ and hybridized on the miRCURY LNA™ microRNA Array 7th Gen (Exiqon, Denmark) using a Tecan HS4800™ hybridization station (Tecan, Austria). After hybridization the microarray slides were scanned using the Agilent G2565BA Microarray Scanner System (Agilent Technologies, Inc., USA) and the image analysis was carried out using the ImaGene® 9 (miRCURY LNA™ microRNA Array Analysis Software, Exiqon, Denmark).

### Quantitative PCR validation

To confirm the microarray results, during validation stage, the quantitative real-time PCR was performed. qPCR analysis was carried out using ExiLENT SYBR Green master mix (Exiqon, Denmark) on CFX96™ Touch Real-Time PCR Detection System (Bio-Rad, Germany) with custom selection of miRCURY LNA™ primers in Pick-&-Mix, 96 well Ready-to-Use formats. The PCR reactions for each miRNA were run in duplicate and the results were averaged over these analyzes. The normalized relative expression level of the genes of interest was calculated according to the dCt method.

### Data preprocessing and statistical analysis

All data analysis and interpretation were performed by us. Microarray data normalization was performed to minimize differences between the colors in an intensity-dependent manner using Lowess (Locally Weighted Scatterplot Smoothing) regression algorithm. miRNAs measured in less than 80% of cases were filtered out. Expression data were log2-transformed and compared using one-way analysis of variance. Expression of all remaining miRNAs was compared between the three groups using an analysis of variance (ANOVA) with the Benjamini-Hochberg procedure used to estimate false discovery rates (FDR). To adjust for multiple hypothesis testing false FDR were calculated for all miRNAs with ones characterized by *p* < 0.05 and FDR < 0.15 being considered as significant for the purpose of classifier development and further validation. Additional post-hoc analysis was performed to further clarify the differential expression between the groups.

To provide further insights into tumors biology, miRNA evaluation of microarray samples was also analyzed in gangliogliomas with *BRAFV600E* mutation (5/19). Differential analysis was performed using unpaired t-test with FDR correction.

Expression of miRNAs in the validation cohort was calculated according to the dCt and results were again compared between the groups using one-way analysis of variance, followed by Tukey’s post-hoc test if significant results (*p* < 0.05) were observed in ANOVA. Hierarchical clustering heatmaps were constructed for both the primary and validation groups using Euclidean distance and complete linkage protocol. Similarly, in the validation cohort, a classification model was developed. The missing expression values in validation cohort prior to data mining modeling were imputed using expectation-maximization algorithm with a multivariate normal model. STATISTICA 13 (Statsoft, Tulsa, OK, USA), MultiExperiment Viewer (Dana-Farber Cancer Institute, Boston, MA, USA) and several R packages (caret, mice, RWeka) were used for statistical analysis [16–18].

Complex data-mining analysis was conducted using Waikato Environment for Knowledge Analysis (WEKA 3.9.0, University of Waikato, New Zeeland). Feature selection based on difference significance in differential expression analysis was performed to decrease dimensionality in microarray data. Subsequently, to evaluate miRNAs subsets using classification model induction were assessed in 10 times 10-fold cross-validation manner. Six modeling approaches were tested in profiling stage, J48 algorithm, ZeroR algorithm, JRip algorithm, random tree and support vector machine with radial basis function kernel [19]. In validation stage the best performing approach was used to induct model confirming the observations from profiling stage. The details of methods used in the modeling with their hyperparameters were provided as supplementary material (Additional file 2: Table S2).

A computational analysis was used to identify mRNA targets for final set of selected miRNAs using miRWalk database [20].

## Results

The final cohort was comprised of 46 cases of DNET (WHO grade I), 57 cases of GG (WHO grade II) and 45 cases of PA (WHO grade I) classified according to the criteria of WHO updated classification [1]. Fifty-one and 107 samples were included in profiling and in validation stage respectively. All patients were under 18 years of age at the time of surgery. Median age was 9 years (range: 5 months to 18 years). Profiling and validation GG subgroups were independent set of samples. In the DNET and PA subgroups five and six specimens, respectively, were collected in both sets (Additional file 1: Table S1).

**Fig. 1:**
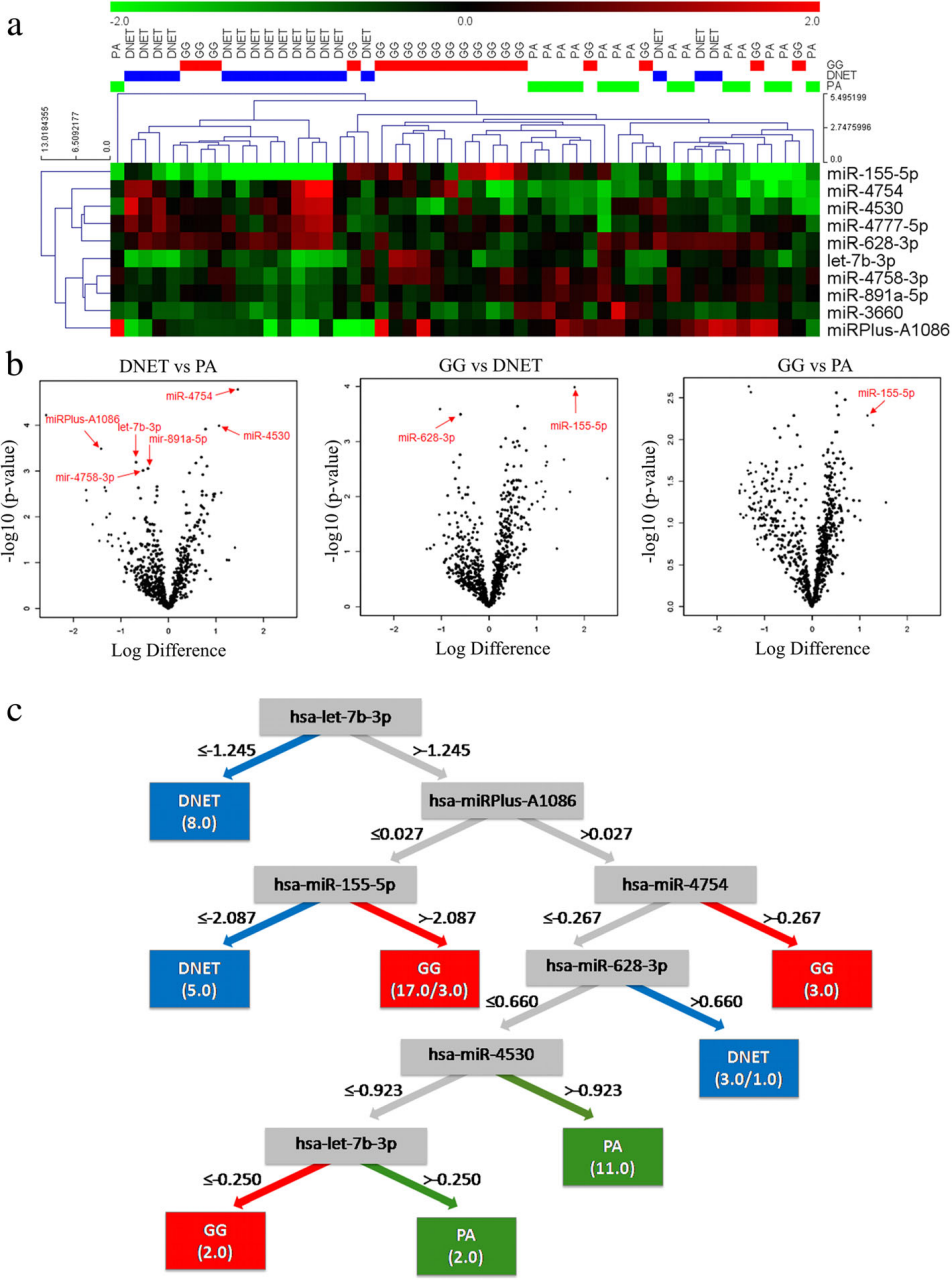
Results of microarray analysis performed in the profiling stage. **a** Heatmap of significantly differently expressed miRNAs between the tumor subgroups; **b** The volcano plots of all three comparisons with miRNAs chosen for validation stage indicated by their labels; **c** The best performing model achieved by application of J48 algorithm on the set of miRNAs selected by significance-based method. *DNET* dysembryoplastic neuroepithelial tumor*, GG* ganglioglioma, *PA* pilocytic astrocytoma

The array quality assessment showed successful labeling for the control spike-in oligonucleotides in the expected range and normalization of the quantified signals using the global LOWESS regression algorithm was performed. Filtration of expression data of 51 microarray samples showed 782 miRNAs eligible for detailed analysis (Additional file 3: Table S3). Differential expression analysis performed in ANOVA showed 10 miRNAs meeting the prespecified criteria significance in tumor subgroups differentiation (Fig. 1a, Additional file 4: Table S4). Pairwise comparisons of the three analyzed groups: DNET, GG and PA showed the DNET tumors to be the most divergent with the largest number of miRNAs differing from either of the two comparative groups (Fig. 1b).

The results presented as the weighted average of 10 random 10-fold cross-validation of modeling for each feature set indicted the J48 algorithm with significance-based feature selection as the method mostly resilient to overfitting (Table 2).

**Table 2:** Weighted area under ROC curve for different feature selection and classifier induction methods showed J48 with significance-based feature selection as the one of the best performing and immune to overfitting modeling approaches. Significance filter was chosen to be preferable based on the highest average across multiple feature selection methods. J48 has been chosen as the best method based on the highest average across all methods

Dataset	J48	ZeroR	Jrip	Decision Stump	Random Tree	SVM	Average
Full dataset	0,59	0,50	0,63	0,66	0,59	0,61	0,60
Significant miRNAs	0,71	0,50	0,71	0,60	0,67	0,50	0,62
Support vector machine-based attribute evaluator	0,66	0,50	0,62	0,49	0,61	0,50	0,56
TTP	0,55	0,50	0,52	0,49	0,53	0,51	0,52
Average	0,63	0,50	0,62	0,56	0,60	0,53	

*SVM* Support vector machine with radial basis function kernel, *TTP* Targeted projection pursuit

The final model on microarray data showed accuracy of 92.2% with weighted area under the ROC curve of 0.971 (*p* < 0.01, Fig. 1c).

Selective miRNA analysis performed for five GGs carrying *BRAFV600E* mutation presented miRNAs which were significantly differentially expressed and none of those were used for final classification (Additional file 5: Table S5).

Proposed approach has confirmed the potential for a miRNA-based classifier which we attempted to calibrate on a separate validation cohort. Validation samples underwent expression measurement using a pick-and-mix array consisting of 8 miRNAs identified using microarray expression profiling and that were crucial for the best model development. Additionally, five normalizers were selected as the most stable reference miRNAs with the NormFinder algorithm [21] (Table 3).

**Table 3:** miRNAs used for qPCR validation of microarray data

No	miRNA	Sequence	Assay symbol
Differential Expression			
1	miR-155-5p	UUAAUGCUAAUCGUGAUAGGGGU	204,308
2	miR-4754	AUGCGGACCUGGGUUAGCGGAGU	2,107,017
3	miR-4530	CCCAGCAGGACGGGAGCG	2,105,012
4	miR-628-3p	UCUAGUAAGAGUGGCAGUCGA	206,057
5	let-7b-3p	CUAUACAACCUACUGCCUUCCC	205,653
6	miR-4758-3p	UGCCCCACCUGCUGACCACCCUC	2,118,014
7	miR-891a-5p	UGCAACGAACCUGAGCCACUGA	204,220
8	miRPlus-A1086	UAGUGCCGUGGUCCUUUUGGC	169,416
Stable Expression			
1	miR-500a-5p	UAAUCCUUGCUACCUGGGUGAGA	204,794
2	miR-451b	UAGCAAGAGAACCAUUACCAUU	2,103,713
3	miR-514b-3p	AUUGACACCUCUGUGAGUGGA	2,108,297
4	miR-1293	UGGGUGGUCUGGAGAUUUGUGC	2,110,424
5	miR-1226-3p	UCACCAGCCCUGUGUUCCCUAG	2,102,736

**Fig. 2:**
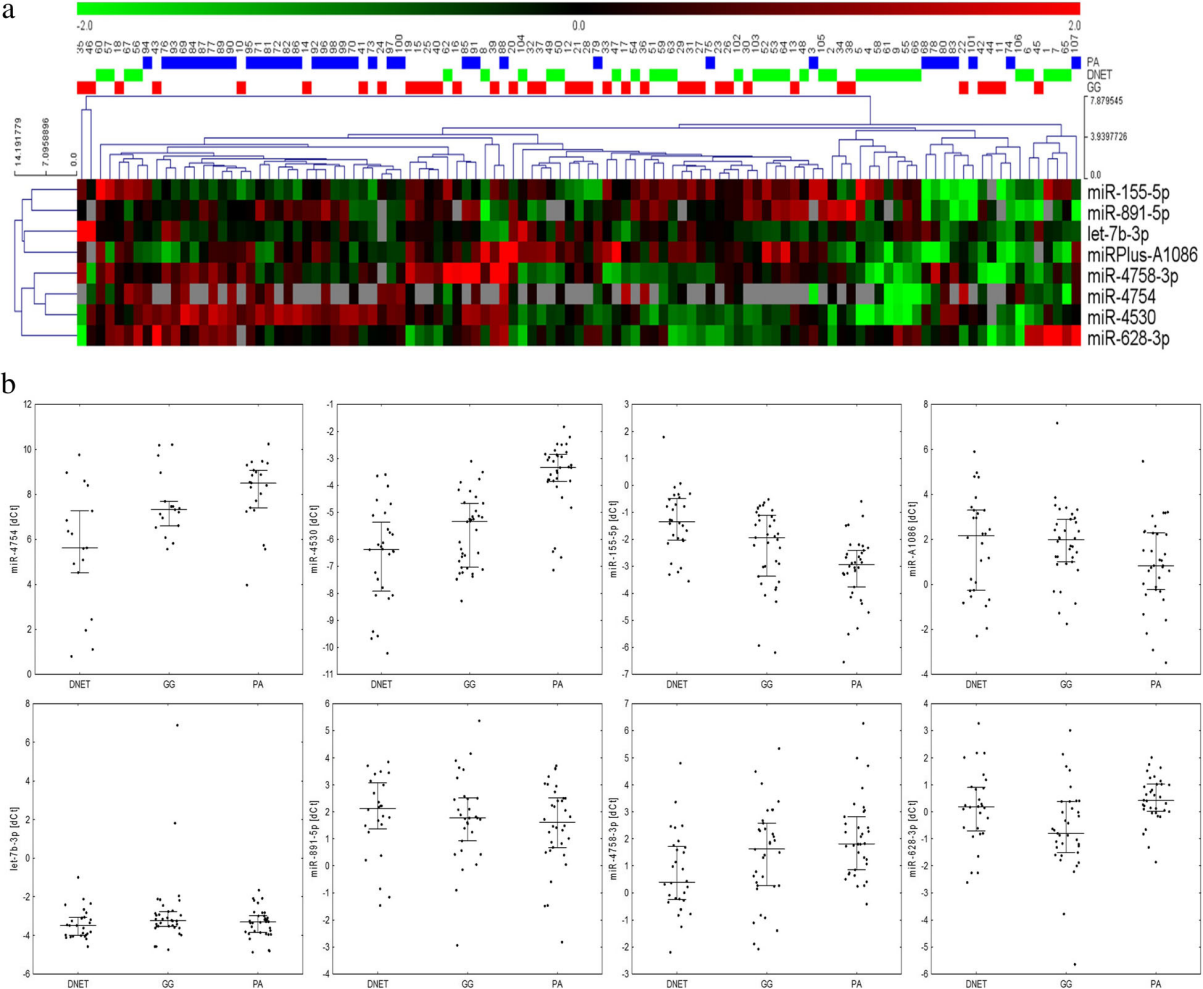
Results of analysis performed in the validation stage. **a** Heatmap of the best miRNA qualifiers differentiating between the tumors subgroups validated by using qPCR; **b** Dot plot showing the relationship between the expression of selected miRNAs and histopathological type of tumor. *DNET* dysembryoplastic neuroepithelial tumor, *GG* ganglioglioma, *PA* pilocytic astrocytoma

Results of the qPCR validation also showed that all three types of analyzed tumors exhibit distinct miRNA expression profiles (Fig. 2, Additional file 6: Table S6). Pairwise comparisons of the miRNAs showed that the differences in miRNA expression in the validation analysis were in line with those observed in the profiling experiment (Additional file 7: Table S7). Significant differences between the groups were identified for miR-4754, miR-4530, miR-155-5p, miR-4758-3p and miR-628-3p. These results confirmed the differences noted in the profiling experiment: miR-4754 showed significantly higher expression in the DNET group than in PA; miR-4530, miR-155-5p and miR-628-3p had the lowest expression in PA, DNET and GG tumors respectively. However, expression of miR-4758-3p was the highest in the DNET samples, which was not a direct confirmation of the microarray experiment, as this group showed the lowest expression previously. The expression of miR-4754 at low level in 55 out of 107 samples rendered it useless for the purpose of developing a classification model.

**Fig. 3:**
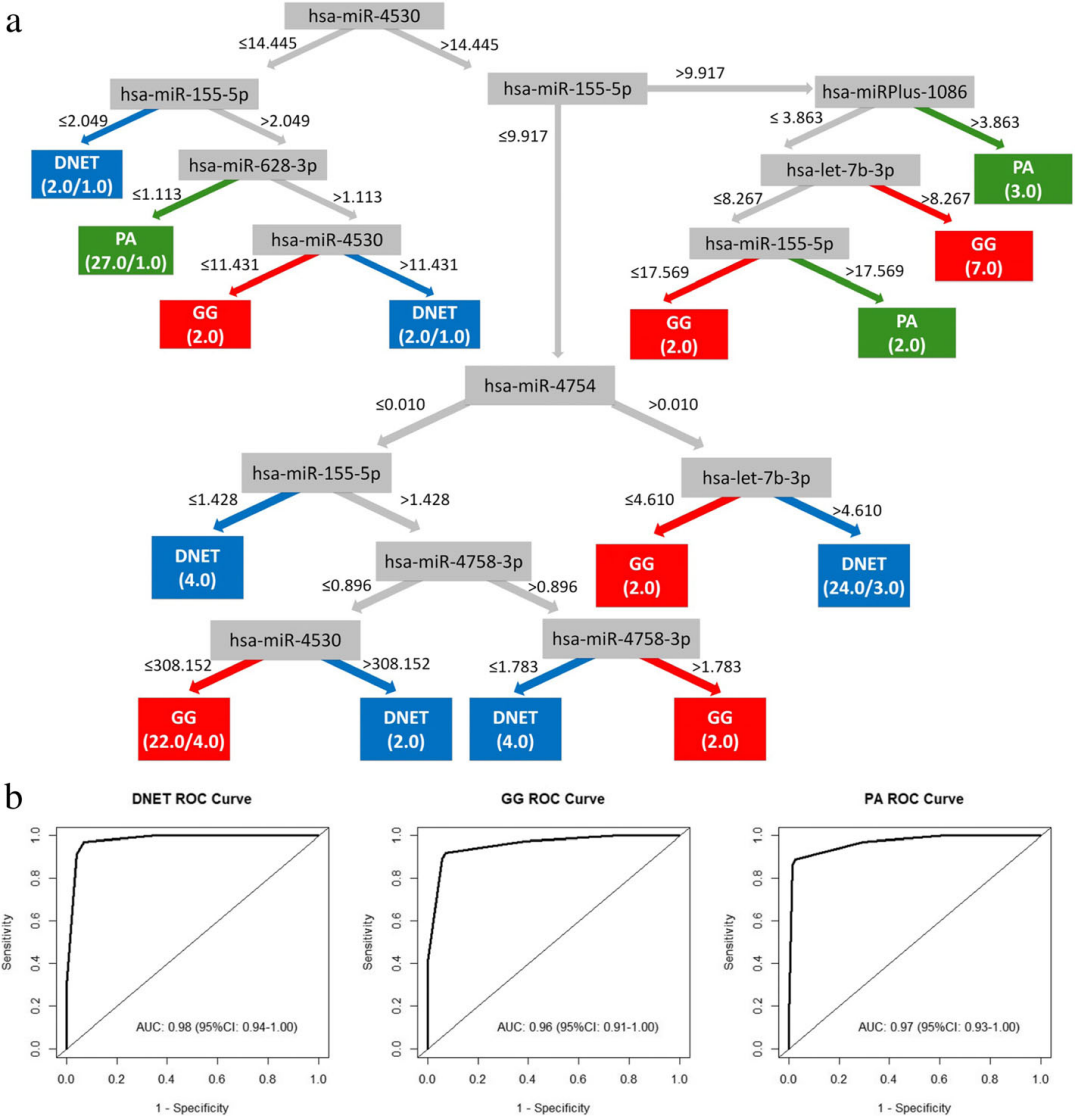
Final decision tree developed on qPCR validation dataset. **a** The structure of best performing expression-based decision tree for differentiation of samples; **b** The predictive abilities of the model by ROC analysis. Class probability estimates based on observed relative class frequencies at the leaf nodes were used for the development of ROC curves. *AUC* area under the ROC curve, *DNET* dysembryoplastic neuroepithelial tumors, *GG* gangliogliomas, *PA* pilocytic astrocytomas, *ROC* receiver operating characteristic curve

Classification model developed on the expression of selected miRNAs for qPCR validation showed the accuracy of 90.7% and weight AUC of 0.97 in validation on training set with possibility of developing an accurate decision tree and was comparable to the results obtained on the profiling stage of analysis (Table 4, Fig. 3).

**Table 4:** miRNA expression fold changes achieved on profiling and validation set of samples for the three analyzed subgroups

miRNA	PA vs. DNET	DNET vs. GG	PA vs. GG
Profiling set FC			
hsa-miR-4754	0,365,273,252	1,734,884,736	0,633,706,989
hsa-miR-4530	0,486,237,157	1,884,327,942	0,91,623,026
hsa-miR-155-5p	1,544,860,921	0,310,106,478	0,479,071,379
hsa-miRPlus-A1086	2,919,325,657	0,563,816,578	1,645,964,203
hsa-let-7b-3p	1,670,915,943	0,631,840,741	1,055,752,768
hsa-miR-891a-5p	1,364,996,166	0,844,233,833	1,152,375,946
hsa-miR-4758-3p	1,462,678,698	0,72,361,195	1,058,411,786
hsa-miR-628-3p	0,821,392,107	1,471,905,349	1,209,011,436
Validation set FC			
hsa-miR-4754	0,142,744,049	12,14,192,594	1,733,187,674
hsa-miR-4530	0,071030983	4,177,155,132	0,296,707,437
hsa-miR-155-5p	3,883,969,214	0,358,060,831	1,390,697,245
hsa-miRPlus-A1086	1,544,571,733	1,576,092,261	2,434,387,555
hsa-let-7b-3p	0,989,336,539	1,189,024,878	1,176,345,758
hsa-miR-891a-5p	240,712,865	0,766,431,411	1,844,899,007
hsa-miR-4758-3p	0,392,739,993	1,189,309,823	0,467,089,532
hsa-miR-628-3p	0,597,175,923	0,418,895,902	0,250,154,547

*DNET* Dysembryoplastic neuroepithelial tumor, *FC* Fold change, *GG* Ganglioglioma, *PA* Pilocytic astrocytoma

Performed here in silico prediction of miRNA-mRNA interactions showed *LNPK* and *LCOR* targets as targeted by more than one of miRNAs (Additional file 8: Table S8). In case of predicted targets, 6 of 8 selected miRNAs were expected to target *ZBTB20*, *LCOR*, *PFKFB2*, *SYNJ2BP* and *TPD52* genes (Additional file 9: Table S9).

## Discussion

Here we studied the miRNA expression in the pediatric glial and mixed glioneuronal tumors. The comparison of dysembryoplastic neuroepithelial tumor, ganglioglioma and pilocytic astrocytoma gave us the information concerning molecular differences detected on the miRNA level. Bioinformatic analysis revealed a number of miRNAs differently expressed in each tumor type with the largest number of miRNAs differentiating DNETs from the two comparative groups. Noteworthy, our models could be used to differentiate the type of tumor without comparison to normal tissue. This is because the miRNAs of stable expression were used as comparators, thus their measurement and subsequent use in normalization is required.

At present little is known about significance of miRNA expression in pediatric glial and mixed tumors and there are scanty data concerning detailed expression analysis of such cases. Previously published miRNA profiling studies were performed on small numbers of cases, which frequently mixed adult and pediatric samples [12, 22–27] (Table 5). Additionally, the analyses performed so far mainly focused on the differences between normal, peritumoral and tumor tissue [22–24]. Such approaches were most probably a consequence of low frequency of mixed glioneuronal tumors occurring in pediatric population.

**Table 5:** Review of the literature data concerning microRNA analyses in glioneuronal tumors

No of samples	Tumor type	Age category	Summary	Ref.
41	mixed LGG	C/A	61 differently expressed miRNAs on profiling study (*n* = 41); the same samples (*n* = 19) used for validation of miR-129-2-3p, miR-219-5p, miR-338-3p, miR-487b, miR-885-5p, miR-323a-3p, miR-4488 and miR-1246; expression of miR-487b from cluster 14q32.31 variably underexpressed in pediatric glioma lines	[24]
34	GG	C/A	evaluation of inflammation-related miR-21, miR-146, miR-155; miR-146 contributed to epileptogenic network	[25]
29	GG	C/A	66 miRNAs differently expressed on profiling study (*n* = 9); validation (*n* = 20) of miR-217 showed relationship between miR-217 and ERK1/2	[22]
5	DNET	C	120 differently expressed miRNAs on profiling study (*n* = 5); the same samples used for validation aof down-regulated miR-3138 and overexpressed miR-1909	[23]
43	PA	C/YA	31 differently expressed miRNAs on profiling study (*n* = 43); validation of miR-21, miR-124 and mir-129 (*n* = 9)	[12]
99	mixed LGG	C/A	miR-519d and miR-4758 upregulated in GGs compared to control tissue, DNET and other gliomas	[50]

*A* Adult, *C* Children, *DNET* Dysembryoplastic neuroepithelial tumor, *GG* Ganglioglioma, *LGG* Low grade glioma, *PA* Pilocytic astrocytoma, *YA* Young adult

We analyzed miRNA profiles of a large number of pediatric samples and confirmed their efficient differentiation on the basis of miRNA expression levels. The decision tree modeling algorithm used by us reached an AUC value 0.97 both in profiling and validation set of samples, which indicates for high discriminatory power of selected miRNAs. These findings indicated that the expression of miRNAs is representative for histopathological subtypes of analyzed tumors of similar origin. Correct classification of glial and glioneuronal pediatric tumors with accuracy of 92.2 and 90.7% on array and validation stage respectively demonstrated high accuracy of the proposed model by combining the expression levels of 8 miRNAs.

Although for some of these miRNAs the relationship to brain tumor formation was mostly unknown, among them there were also extensively studied molecules such as highly conserved miR-155 with confirmed altered expression in various types of cancers [25, 28–30]. It has an assigned oncogenic function, also confirmed in gliomas with plausible role in tumor development and progression [31]. Higher levels of miR-155 were observed in tumor specimens and peritumoral tissue in gliomas of different grade and histopathological features [29]. Methylation of gene promoter region reduced the miRNA expression and was correlated with longer survival of patients with glial tumors [30]. It was also considered as a potential molecular treatment target due to the correlation with WNT pathway [31]. On the basis of up-regulation in macrophages, monocytes, and microglia in response to pro-inflammatory stimuli the molecule was also regarded as an important factor involved in inflammatory processes [32, 33].

Our observation of higher expression levels of miR-155-5p in DNETs and GGs indicated for its potential role in tumors coupled with neuronal dysfunction. Presence of that miRNA in the proposed expression-based decision tree for differentiation of mixed tumors also strongly indicates for miR-155-5p significance in brain tumor biology. Such observations suggest possibility of new treatment modalities influencing miRNA expression especially in patients with drug-resistant epilepsy and higher levels of miR-155 [25, 34].

Furthermore miR-155-5p was one out of four miRNAs positively validated by us, with a putative relation to developmental and/or inflammatory processes. In that context the value of tumors inflammatory microenvironment seems to be important during the development of glial and mixed neuronal-glial tumors. Up to now such association was underlined by Prabowo et al. in their analysis of three miRNAs involved in the regulation of the pro-inflammatory pathways, miR-146a, miR-21, and miR-155 [25]. The observation concerning relationship between miRNA expression and developmental delay, including intellectual disability and seizures, was noted also by Carvalheira et al. on the basis of molecular analysis of patients with chromosome 19 rearrangements [32]. They indicated that miR-4754, which has also been selected in our study, could be related to brain development mainly on the basis of its possible target genes *JMY*, *CNTN3* and *EYA4* [32].

Performed here computational in silico analysis of miRNA-mRNA network also showed connection with genes expressed during human brain development, *LNPK* and *ZBTB20*. Two out of 8 selected miRNAs have shown to target *LNPK*, encoding the endoplasmic reticulum junction stabilizer lunapark, gene with assigned role in neurodevelopmental disorders and plausible contribution to the epilepsy [35]. In turn transcriptional repressor *ZBTB20* had confirmed function in neural precursor cells [36]. Moreover for *ZBTB20* and *PFKFB2* genes plausible link between genes alteration and glioma proliferation and invasion was underlined, especially in the context of glioma-associated cells [37, 38]. Similar function was assigned to another gene selected in our analysis, miR-4530 which plausibly contributes to the promotion of tumor angiogenesis in endothelial cells and possibly to tumor malignant transformation [39–41]. Pro-angiogenic function in tumor formation was also reported for oncomiR-891a-5p which has a suspected pro-tumorigenic role [42, 43]. Reports exist for involvement of miR-628-3p, miR-4758-3p, let-7b-3p and miRPlus-A1086 in pro-tumoral networks. Up-regulation of miR-628-3p was observed in different types of extracranial tumors in adult and pediatric patients [44]. What is more, the molecule’s altered expression was observed in patients with Huntington’s disease and it could be interpreted as an indirect evidence for its involvement in brain function and development [45–48]. let-7b has suspected tumor suppressor function in gliomas and low expression of the let-7 family members may be responsible for the poor prognosis of brain tumors patients through disturbed inhibition of *KRAS*, *HMGA2* and *MYC* oncogenes [49–51]. Our study indicated for following potential targetable biomarker, *TPD52* which overexpression were noted in many cancer types including brain tumors [52, 53]. Here we showed that in case of predicted targets, 6 of 8 selected miRNAs were expected to target *TPD52* gene during in silico prediction.

Ultimately, the limited information concerning miRPlus-A1086 showed their relationship to treatment modalities in prostate cancer and melanoma [52, 54].

Taking the above into consideration, it seems that all the miRNAs selected in the current study have previously been reported as functional in cancers, what suggests that the selection method detected not only the most differentially expressed molecules but also identify biologically important miRNAs [45–48, 55].

Recent analysis published by Bongaarts et al. showed miR-4758 as up-regulated in GG compared to control cortex with AUC reaching 0.837. The differences were not significant compared with PA and confirmed for DNETs [56]. Here we revealed the classifiable utility for miR-4758-3p together with remaining 7 miRNAs for pediatric neuronal and mixed neuronal-glial brain tumors with high reproducibility of results. This was likely made possible due to the number of genes selected for the proposed model, a large number of samples included in the analysis, as well as limiting the sample pool only to the pediatric population. It enables us to define characteristic miRNA profile of different tumors of neuroepithelial origin with decision tree showing tremendous performance on training set and in leave-one-out cross-validation.

## Conclusions

Here we showed that distinct miRNA expression pattern is a specific feature of different pediatric low grade gliomas. A proposed classification model based on the set of miRNA may be a useful classification tool in cases that are difficult to distinguish by classical histopathological examination. Known biological stability of miRNA indicates for usefulness of proposed decision tree in gangliogliomas, dysembryoplastic neuroepithelial tumors and pilocytic astrocytomas classification by using qPCR method, which is widely recognized as the gold standard for analyzing the expression of miRNAs.

The study has also a potential to increase our understanding of the molecular mechanisms of brain tumors development. Additionally, our observations suggest that histological transformation between glial and mixed pediatric tumors could be a consequence of new alterations occurring during cancerogenesis which lead to morphogenetic changes regardless of core molecular alterations.

## Additional files

Additional file 1: **Table S1.** Tumor detailed characteristics. * indicates samples used in profiling experiment, *^ indicates samples included both in profiling and validation cohort. *DNET* dysembryoplastic neuroepithelial tumor, *GG* ganglioglioma, *PA* pilocytic astrocytoma. (XLSX 16 kb)

Additional file 2: **Table S2.** Configuration of hyperparameters set a priori for modelling in WEKA software. (XLSX 9 kb)

Additional file 3: **Table S3.** Log2-transformed expression data of samples assessed using microarrays in profiling stage. *DNET* dysembryoplastic neuroepithelial tumor, *GG* ganglioglioma, *PA* pilocytic astrocytoma. (XLSX 1599 kb)

Additional file 4: **Table S4.** Results of microarray data pairwise comparisons between the three groups. *AVG* average, *DNET* dysembryoplastic neuroepithelial tumor, *FC* fold change, *FDR* false discovery rate, *GG* ganglioglioma, *PA* pilocytic astrocytoma, *SD* standard deviation. (XLSX 182 kb)

Additional file 5: **Table S5.** List of miRNAs differentially expressed in *BRAFV600E* mutated and wild type cases (GG12, GG25, GG28, GG33, GG55). Negative fold change indicates downregulation in mutated cases. miRNAs selected for validation has been appended to the Table. *FC* fold change, *FDR* false detection rate. (XLSX 11 kb)

Additional file 6: **Table S6.** Transformed delta Ct (2^-dCt) values of miRNAs expression obtained in validation stage. (XLSX 25 kb)

Additional file 7: **Table S7.** Results of pairwise comparisons between the three groups in qPCR validation. *AVG* average, *DNET* dysembryoplastic neuroepithelial tumor, *FC* fold change, *FDR* false discovery rate, *GG* ganglioglioma, *PA* pilocytic astrocytoma, *SD* standard deviation. (XLSX 11 kb)

Additional file 8: **Table S8.** Genes targeted by selected 8 miRNAs along with the list of targeting miRNAs. Only experimentally validated targets have been summarized in this table showing results of in silico analysis. (XLSX 11 kb)

Additional file 9: **Table S9.** Genes targeted by selected 8 miRNAs along with the list of targeting miRNAs. Experimentally validated and predicted targets have been summarized in this table showing results of in silico analysis. (XLSX 164 kb)

## Data Availability

The datasets used and/or analyzed during the current study are available from the corresponding author on reasonable request.

## Abbreviations

AUC: Area under the receiver operating characteristic curve
AVG: Average
DNET: Dysembryoplastic neuroepithelial tumor
FC: Fold change
FDR: False discovery rate
GG: Ganglioglioma
miRNA: MicroRNA
PA: Pilocytic astrocytoma
qPCR: Quantitative real-time polymerase chain reaction
ROC: Receiver operating characteristic curve
SD: Standard deviation
SVM: Support vector machine with radial basis function kernel
TTP: Targeted projection pursuit
